# Celecoxib-induced acute generalized exanthematous pustulosis: uncommon and under-recognized side effect

**DOI:** 10.17179/excli2023-6809

**Published:** 2024-01-24

**Authors:** Abul Hasan Shadali Abdul Khader, Meenu Singh

**Affiliations:** 1 University of Utah Medical Center, Salt Lake City, Utah, USA

**Keywords:** celecoxib, NSAIDs, dermatology, adverse drug reactions

## Abstract

Celecoxib, a selective COX-2 inhibitor, and non-selective anti-inflammatory drug, is commonly prescribed as the first-line analgesic for osteoarthritis, rheumatoid arthritis, and certain acute pain cases. It is mainly preferred for its lower risk of gastrointestinal adverse effects. However, it also carries risks, including renal and liver toxicity, anaphylaxis, and Stevens-Johnson syndrome. A rare but severe cutaneous adverse reaction associated with celecoxib is Acute Generalized Exanthematous Pustulosis (AGEP), characterized by extensive nonfollicular sterile pustules on an erythematous background, fever, and neutrophilic leukocytosis. AGEP is a rare condition with an incidence rate of 1-5 cases per million per year in the general population. It is primarily triggered by drugs, with antibiotics accounting for over 90 % of cases. Here, we present the case of a 44-year-old female who presented with a sudden, rapidly progressive, painful, pruritic rash all over her body with associated leukocytosis. A skin biopsy confirmed the presence of a pustular rash. The patient reported taking Celebrex (celecoxib) for worsening arthritis two weeks prior to symptom onset. The patient was diagnosed with Celecoxib-induced AGEP based on clinical and histopathological features. Treatment involved steroid therapy and discontinuation of NSAIDs (non-steroidal anti-inflammatory drugs). Encouragingly, the patient's rash improved within three days. Our case report aims to raise awareness of AGEP as a side effect of NSAIDs. Although AGEP is not typically serious, it can be fatal in elderly patients. Therefore, prompt identification and immediate cessation of the culprit drug is crucial.

## Introduction

Acute generalized exanthematous pustulosis (AGEP) is an uncommon but severe cutaneous reaction. Most cases are triggered by medications, with antibiotics accounting for the majority (Lee et al., 2016[5]). Celecoxib, a non-steroidal anti-inflammatory drug (NSAID) selectively inhibiting cyclooxygenase-2 (COX-2), is widely used for pain relief in arthritis due to its lower rate of gastrointestinal irritation (Goeschke and Braathen, 2004[4]).While cutaneous adverse events are typically benign, rare severe reactions have been documented (Marquès et al., 2003[6]). To date, only five cases of celecoxib induced AGEP have been reported in the literature (Britschgi et al., 2001[3]; Marquès et al., 2003[6]; Yang et al., 2004[11]; Shin et al., 2011[9]; Lee et al., 2016[5]). However, AGEP cases remain underreported due to their self-limiting nature in most cases and the clinical similarities with other drug eruptions (Moore et al., 2023[7]). In this report, we present a case of celecoxib-induced AGEP in a middle-aged female, contributing further insights to this field of research.

## Case Report

A 44-year-old female presented to our hospital with a sudden onset of a painful rash that spread across her entire body over a week prior to presentation. The rash progressed quickly, causing intense pain of 10/10 severity, in addition to pruritus. The patient denied any presence of fever or mucosal involvement. She reported that she had been taking a new anti-inflammatory drug (Celebrex-200 mg) for her worsening arthritis symptoms, about 2 weeks prior. Her medical history included osteoarthritis and hypertension. She had no personal history of eczema/psoriasis, but did have a family history of psoriasis.

**Figure 1:**
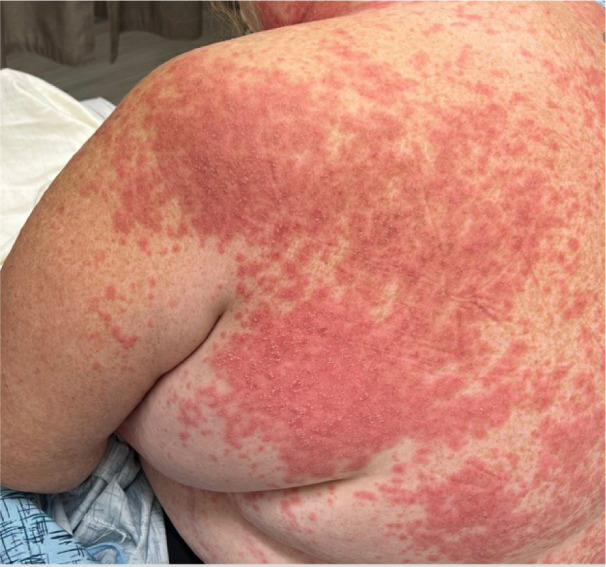
AGEP Clinical Image showing non-follicular pustules in an erythematous background

**Figure 2:**
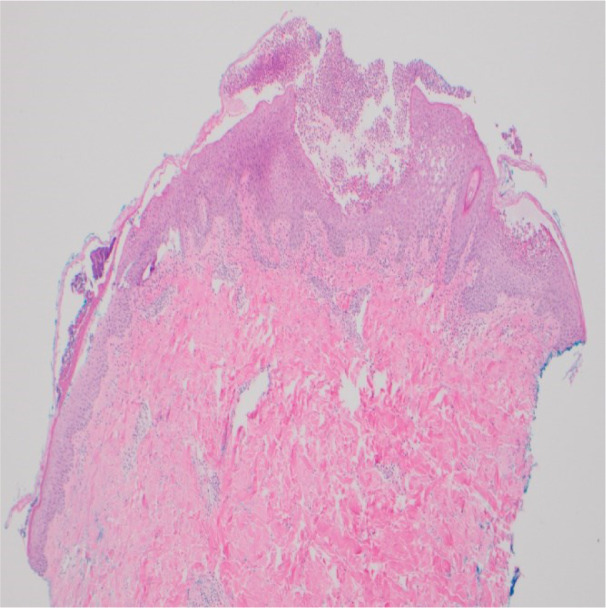
Histopathological image showing collections of large sub-corneal neutrophils

Upon examination, the patient was afebrile (36.9 °C) with stable vital signs. Physical examination was remarkable for numerous erythematous papules and plaques with overlying desquamation, along with non-follicular pustules that were distributed across the neck, back, chest, abdomen, and upper extremities (Figure 1(Fig. 1)). Notably, the face, lower extremities, and mucosal areas were largely spared. Laboratory investigations showed elevated leucocyte count of 13,900/microL with neutrophilia (4,500 to 11,000 WBCs/microL), normal absolute eosinophil count, and elevated CRP of 1.3 mg/dL (0.0-0.8 mg/dL). The comprehensive metabolic panel including liver and renal function tests were unremarkable. Hepatitis and HIV serologies were also negative. A punch biopsy from the lesion in the right upper back was obtained. Histopathological examination confirmed the presence of large subcorneal neutrophilic pustules with focal areas of neutrophilic spongiosis with dermal superficial perivascular lymphocytic infiltrate. Furthermore, mild eosinophilic spongiosis and acanthosis were also noted in epidermal layers (Figure 2(Fig. 2)). PAS staining gave negative results, excluding infectious causes. Direct immunofluorescence testing for antibodies (IgG, IgG4, IgM, IgA, and C3) yielded negative results.

These histopathological findings raised several differential diagnoses for the pustular rash that includes pustular psoriasis, acute generalized exanthematous pustulosis, and a superficial variant of pemphigus. Based on the clinical history, recent drug exposure, and histopathological evidence, a diagnosis of Celebrex-induced AGEP was established. The patient's use of NSAIDs was discontinued immediately, and she was treated with topical triamcinolone, and high-dose prednisone as treatment. Pain was managed with Tylenol and oxycodone. Encouragingly, the patient's rash showed marked clinical improvement in 3 days, with complete resolution in a week.

## Discussion

First described by Baker and Ryan in 1968, as 'pustuloses exanthématiques aiguës généralisées', and later term AGEP was coined by Beylot in 1980, to describe an acute noninfectious pustular eruption mainly induced by drugs with no history of psoriasis with a typical histology (Beylot et al., 1980[2]). More than 90 % of the AGEP cases are caused by drugs, mainly antibiotics like beta-lactam antibiotics and macrolides. Other drugs reported include hydroxychloroquine, antifungal, antiviral, and antiparasitic drugs. Other triggers include infections like chlamydia, mycoplasma, CMV, and EBV. Recently, vaccinations with Flu and COVID-19 vaccine have also been reported (Parisi et al., 2023[8]). Celecoxib, a COX-2 inhibitor and NSAID, is recommended by FDA for inflammatory pain caused by rheumatoid arthritis and osteoarthritis (Goeschke and Braathen 2004[4]). It is commonly used due to its lower rate of gastrointestinal irritation compared to non-selective NSAIDs. Celecoxib's cutaneous adverse effects include urticaria, anaphylaxis, erythema multiforme, fixed drug eruption, and SJS/TEN (Shin et al., 2011[9]). AGEP has been reported in 6 cases in association with celecoxib. In some cases, analgesics like ibuprofen, Piroxicam, lornoxicam have also been reported (Parisi et al., 2023[8]) to cause AGEP. An overview of all celecoxib induced AGEP is shown in Table 1(Tab. 1) (References in Table 1: Britschgi et al., 2001[3]; Goeschke and Braathen, 2004[4]; Lee et al., 2016[5]; Marquès et al., 2003[6]; Shin et al., 2011[9]; Yang et al., 2004[11]).

AGEP is more common in women with an average age of 56 years. AGEP usually presents within 24-48 h after taking the drug. However, latency time with celecoxib can take between 3-14 days, like in our case where the patient experienced symptoms after 7 days. Initially, AGEP starts as erythematous patches/plaques in large skin folds, followed by the eruption of multiple punctate, non-follicular, sterile pustules with subsequent typical desquamation within 15 days. Lesions typically appear in intertriginous areas before spreading to other parts of the body. Mild mucosal involvement (lips and buccal mucosa) occurs in about 20 % of cases. The lesion is often accompanied by pruritus and systemic symptoms, including fever and leukocytosis. All Celecoxib-induced AGEP cases are associated with fever, except for our case which was afebrile (with the max temperature of 36.9 °C) along with severe pain in the rash. AGEP is associated with hepatic, renal, or pulmonary involvement (<20 %) in some cases, but cases due to celecoxib did not have any reported systemic involvement (Parisi et al., 2023[8]).

The pathogenesis of AGEP is not fully understood, but it is primarily characterized as a T-cell-mediated type IV hypersensitivity reaction with neutrophilic inflammation. The drug-specific CD4+ and CD8+ cells produce cytotoxic proteins (perforin, granzyme B, and Fas ligand) that cause keratinocyte apoptosis, resulting in vesicles that become sterile pustules as neutrophils are recruited via CXCL8, IFN-gamma, and IL-8. The innate immune system also plays a role through cytokines IL-17 and IL-22, promoting downstream IL-8 secretion and recruitment of neutrophils (Moore et al., 2023[7]).

Diagnosis of AGEP is based on clinical and histologic findings. Rapid onset, exposure to offending medication, and resolution on discontinuation are the main clues for diagnosis (Moore et al., 2023[7]; Parisi et al., 2023[8]). EuroSCAR has a validated score for diagnosis based on morphology, clinical course, and histology. Our case had an AGEP score of 10, indicating a definite AGEP. Supportive diagnostic tests include drug patch testing, prick testing and lymphocyte transformation testing. Drug patch testing has sensitivity of 50-58 % and is recommended 4 weeks after the resolution of AGEP (Shin et al., 2011[9]; Parisi et al., 2023[8]). Histopathologic findings with AGEP involve non-follicular spongiform pustules in the epidermis (intracorneal/subcorneal), necrotic keratinocytes, mixed inflammatory dermal and interstitial infiltrates, and edema of the papillary dermis with neutrophilic and eosinophilic perivascular infiltrates. AGEP can be distinguished from pustular psoriasis by the lack of dilated blood vessels in the papillary dermis and the presence of necrotic keratinocytes with dermal mixed neutrophilic and eosinophilic infiltrates (Moore et al., 2023[7]; Parisi et al., 2023[8]).

The differential diagnosis of AGEP is broad as shown in Table 2(Tab. 2) (Sidoroff et al., 2001[10]; Parisi et al., 2023[8]). Diagnosis is difficult in some cases, wherein the pustules may coalesce becoming more prominent, leading to superficial skin detachment with gentle pressure (positive Nikolsky sign). Pustular psoriasis (PP) is typically the most difficult to differentiate because of similar pustular and desquamative phases following initiation of medications. However, based on retrospective data, they can be differentiated by clinical history of psoriasis, presence of comorbidities, histopathological findings and cell surface markers. Also, common culprit medications for PP include TNF-α inhibitors, lithium, interferons, antimalarials, and beta-blockers (Parisi et al., 2023[8]).

Treatment for AGEP involves discontinuing the offending drug and providing supportive care with topical steroids, antipyretics, and antihistamines. Systemic steroids may be necessary in severe cases (Parisi et al., 2023[8]). Antibiotics are not required unless there are signs of infection (Shin et al., 2011[9]). Prompt diagnosis and discontinuation of the culprit drug are important to prevent fatalities, especially in elderly patients (Lee et al., 2016[5]). Cyclosporine, Secukinumab, and infliximab are available options for steroid-refractory cases. Symptom resolution typically occurs within 2 weeks of discontinuing the drug. It is crucial to note that AGEP recurs with reintroduction of the culprit drug. The mortality rate for AGEP is below 5 % and is caused by multi-organ dysfunction, disseminated intravascular coagulation, and nosocomial infections rather than cutaneous pathologies. Risk factors for high mortality include multiple comorbidities and extensive cutaneous involvement (Parisi et al., 2023[8]).

## Conclusions

To the best of our knowledge, this is the first case report of Celecoxib induced AGEP in the American literature. With the increasing usage of celecoxib and other non-steroidal anti-inflammatory drugs (NSAIDs) for musculoskeletal pain, it is essential for clinicians to recognize its potential to cause AGEP. Additionally, clinicians should learn to differentiate from other common drug-related eruptions to ensure prompt and effective management in acute care settings.

## Declaration

### Financial interest

None declared.

### Conflict of interest

The authors declare that they have no conflict of interest.

### Acknowledgments

We sincerely acknowledge and thank Dr. Jamie Zussman (Dermatopathologist, University of Utah) for the pathology slides.

## Figures and Tables

**Table 1:**
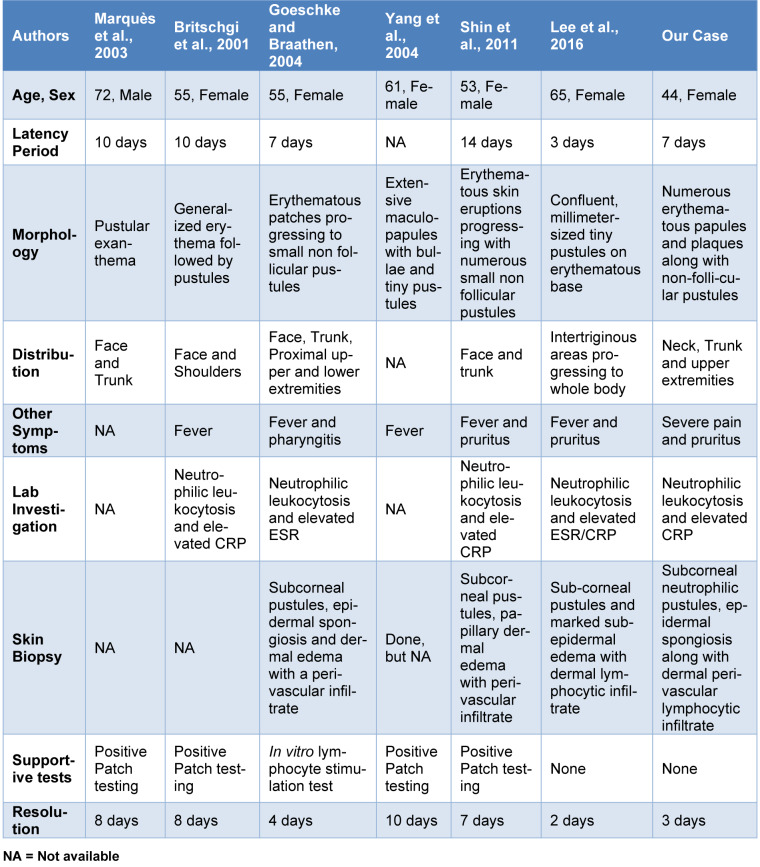
Overview of all Celecoxib-induced AGEP cases

**Table 2:**
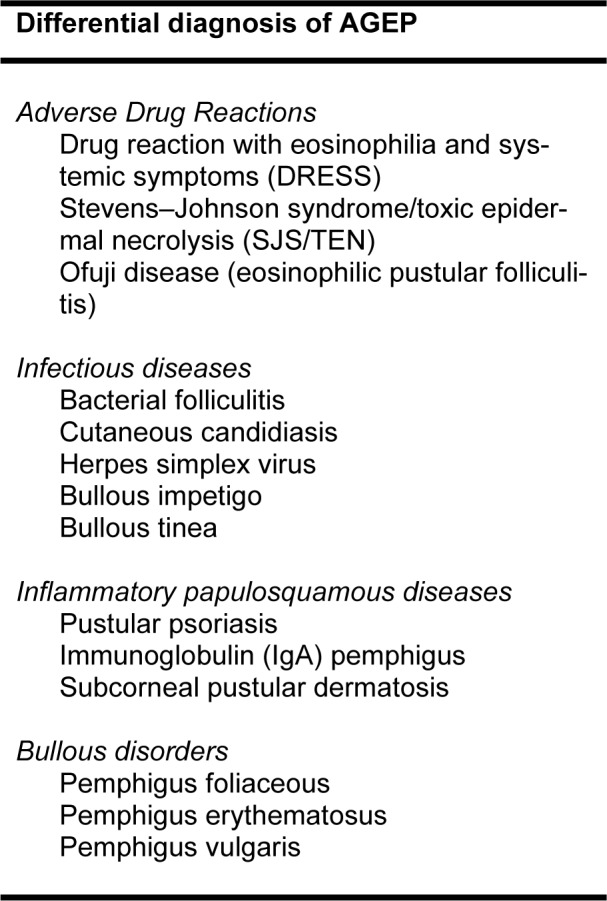
Differential diagnosis of AGEP
